# A comparative analysis of the work environments for registered nurses, nurse aides, and caregivers using the 5th Korean Working Conditions Survey

**DOI:** 10.1186/s12912-022-01120-9

**Published:** 2022-12-13

**Authors:** Sung Shin Kim, Yun Jin Kim, Jun Sik Park, Seoung Hee Ho, Hyosun Kweon, Young-Hyeon Bae

**Affiliations:** 1 Department of Clinical Rehabilitation Research, Rehabilitation Research Institute, Korea National Rehabilitation Center, Seoul, Korea; 2 Department of Healthcare and Public Health, Rehabilitation Research Institute, Korea National Rehabilitation Center, 58, Samgaksan-ro, Gangbuk-gu, Seoul, Republic of Korea

**Keywords:** South Korea, Work environment, Satisfaction, Healthcare worker, Nurses, Caregivers, Aides

## Abstract

**Background:**

Since the quality of work life of healthcare workers is affected by various factors, an improvement in their work environment can reduce the burden on them, increasing their performance. This study aimed to identify the current problems in working environments for registered nurses (RNs), nurse aides (NAs), and caregivers using the 5th Korean Working Conditions Survey (KWCS), presenting measures to improve working conditions by analyzing their predictors: 1) degree of exposure to work-related risk factors (musculoskeletal and mental), 2) working patterns, 3) work-family balance, 4) work situations, and 5) self-rated health.

**Methods:**

The sampling frame was a list of apartment and general survey zones, excluding islands, dormitories, special social facilities, tourist hotels, and foreigner zones, among the total survey zones of the 2010 Population and Housing Census. The KWCS was given to 50,205 participants of various occupations, and responses from 494 RNs, 201 NAs, and 505 caregivers were extracted to compare their 1) degree of exposure to work-related risk factors (musculoskeletal and mental), 2) working pattern, 3) work-family balance, 4) work situations, and 5) self-rated health.

**Results:**

The response rate was 0.449. There were significant differences in all the variables (exposure to musculoskeletal and mental work-related risk factors, working pattern, work-family balance, work situations, self-rated health, and satisfaction with working conditions) among RNs, NAs, and caregivers (*p* < 0.001). The degree of work-related musculoskeletal and mental risk exposure was higher among caregivers and RNs than among NAs; irregular working patterns, challenges with work-family balance, and work environment satisfaction were higher among caregivers than among NAs. In addition, work situations were poorer among caregivers and NAs than among RNs. Self-rated health was the highest among caregivers, followed by RNs and NAs. The most potent predictor of self-rated health was occupation, followed by work environment satisfaction and work-family balance; the most potent predictor of work environment satisfaction was self-rated health, followed by degree of exposure to work-related musculoskeletal and mental risk factors, occupation, work-family balance, work situation, and working patterns.

**Conclusion:**

This study confirmed that a variety of factors influence work environment satisfaction. Thus, practical and realistic measures to improve work environments tailored to each healthcare occupation should be developed at the national and community levels. Further qualitative studies are needed to analyze the work environments of nurses and other care workers in depth.

**Supplementary Information:**

The online version contains supplementary material available at 10.1186/s12912-022-01120-9.

## Background

As a result of changes in population structure and disease patterns, increased awareness of human rights among the public, and changes in social systems, there is a growing demand for healthcare services among consumers. In this context, securing a competitive edge in providing quality healthcare services has become a key issue for healthcare organizations [1]. Registered nurses (RNs) develop a close relationship with patients and provide direct patient care; comprising the largest group of professionals in a hospital, they shape the overall image of the organization, playing pivotal roles in improving the quality of healthcare services and work performance [2].

According to the 2011 Survey on Health Care Level of South Koreans, nursing staff (including nurse aides [NAs]), who account for more than 60% of the healthcare staff, are in charge of observing patients around the clock and providing timely care [3]. While a better social reputation leads to improved nursing performance, they continue to experience stress to improve performance in accordance with social expectations [4], with their stress levels being higher than those in other occupations [5].

Due to the modernization of the nuclear family in modern Korean society and the change in values regarding parental and family care, it is becoming increasingly difficult for families to provide care and support for patients, leading to an increased demand for paid caregivers who can assume this responsibility for them. In response to such demands, a new occupation, titled “caregivers”—paid workers, who are not a family member, providing care for a patient— emerged in South Korea in the early 1980s; as the number of family caregivers is declining, the use of paid caregivers has become more common, with demands continuing to grow [6].

According to a study that investigated 1,584 healthcare facilities nationwide, a total of 17,821 caregivers worked in a hospital, and the caregiver utilization rate in hospitals or higher level healthcare facilities was high, at about 13.2–18.0% in South Korea [7]. However, the caregivers worked under extremely poor conditions, experiencing anxiety about their position, conflict with healthcare workers, low pay, and labor-intensive work [8, 9].

Quality of work life (QWL) is defined as the satisfaction of workers’ basic needs, such as survival and self-realization, through the effective usage of relevant resources in the organization [10]; QWL in healthcare organizations is explained as a combination of the strengths and weaknesses of the work environment [11]. The work performance of healthcare workers can be influenced by macroscopic factors (health management system) [12], microscopic factors (workplace, facility, and environment) [12], and individual factors (lifestyle and health) [12, 13]. Optimizing performance through macroscopic and microscopic changes, as opposed to individual changes, helps reduce the burden on individual workers, improving their performance [14]. Ameliorating such a work environment is the most efficient method for boosting performance at work [15].

In a workplace, the physical environment and facility account for the smallest portion of the total investment (5–7%). However, they affect work performance and welfare, which account for the greatest part of the total cost regarding healthcare life cycle (40–50%) [16–18]. According to the Institute of Medicine, promoting patient safety and enhancing work quality and efficiency in healthcare facilities are dependent on the design of the physical work environment [18]; analyzing the users’ feedback is essential to fostering a successful work environment [19]. Additionally, incorporating workers’ views is crucial when planning, assessing, and improving workplaces to ensure a better work environment that facilitates work and rest [20].

Previous studies have reported that work environment is influenced by work conditions, employment stability, work and financial benefits, positive associations between morale and productivity, equal employment opportunities, human needs and expectations, and the relationship between motivation and leadership [21]. Many studies have identified work environment satisfaction, work interaction, work conditions, compensation, interpersonal relationships, and relationship with and support from the management as the major factors contributing to an effective workplace [22–24].

The predictors of work environment satisfaction include: 1) work life/family life, 2) work design, 3) work situations and work system, and 4) hospital performance (evidence, open system, internal process, and interpersonal relationships) [25]. These factors mutually influence work satisfaction [25–31], practical problems, work performance, relationship with and support from top management, work factors, promotion, pay and compensation, and autonomy [32]. Prior studies that analyzed various factors pertaining to work environment satisfaction reported diverse findings.

In one study, “shared values/organizational atmosphere” was reported as the most important factor [30], while another study stated that cognitive abilities to achieve professional goals, which determine the satisfaction with the healthcare system and quality of care, were more important [28]. A study on nurses showed that interpersonal relationships, occupational values, and self-realization were key factors related to the work environment [24]. In particular, the nursing work environment was linked to the organizational or field atmosphere, bonding among workers, organizational climate and culture, and workers’ maturity. Thus, enhancing the quality of nursing and satisfaction with nursing work may be key predictors of nursing work performance [33].

Analyzing the factors influencing work environment satisfaction is conducive to improving the work environment for healthcare workers. The impact of work environment-related factors on performance and health is dependent on the nature of work and other factors, and can potentially be optimized according to workers’ characteristics and job demands. For example, complex work performance is more likely to be hindered than simple work performance [34, 35] and long working hours per day and per week are associated with work-related musculoskeletal disorders [36]. As shown here, the factors may have varying impacts on the work environment depending on the occupation [37]. Thus, identifying these factors for each specific occupation is crucial for boosting work performance and ameliorating the work environment.

The 5^th^ Korean Working Conditions Survey (KWCS) conducted in 2017 investigated the current working and employment conditions among 50,205 workers in Korea. The KWCS aims to collect baseline data for occupational injury prevention policies by investigating various employment and working environments that affect occupational safety and health. The survey comprises 130 items, including labor intensity, stress, repetitive work, work pattern, emotional labor, education and training, violence/discrimination, job satisfaction, health problems, and exposure to risk factors.

The obtained data from the 5^th^ KWCS has an important significance that it can be used to comprehensively analyze various predictors of work environment satisfaction among care workers, such as RNs, NAs, and caregivers. Thus, this study aimed to identify the current problems with the work environment and develop measures to improve it, while enhancing the conditions for care providers (RNs, NAs, caregivers) using the following data from the 5^th^ KWCS: 1) degree of exposure to work-related risk factors (musculoskeletal and mental), 2) working patterns, 3) work-family balance, 4) work situations, and 5) self-rated health.

## Methods

### Study population

The 2017 KWCS conducted a 1:1 interview with approximately 50,205 workers aged 15 and over in 17 cities and provinces in Korea from July 11 to November 17, 2017, by visiting households. The survey was conducted by professional researchers who had completed interview training. As a survey tool, the Blaise program, a statistical survey system for computer-assisted personal interview (CAPI), developed by Statistics Netherlands, was introduced, and an electronic questionnaire mounted on a tablet PC was used instead of the existing paper survey table [38]. This survey about the various work and employment conditions of workers is administered in person, enabling a comprehensive analysis of the work environments of RNs, NAs and caregivers [39]. For this study, data from 494 RNs (code 2430), 201 NAs (code 2465), and 505 caregivers (code 4211) were analyzed in accordance with the job classification code used in the 50,205 worker of 2017 KWCS.

### Measurement variables

The KWCS was developed based on the European Working Conditions Survey (EWCS) to provide a better working environment for employees through identifying work-related factors and health effects. The external validity was proved using sound sampling procedures. The validity and reliability of KWCS have been verified in previous studies [40, 41]. The validity of the content was assured by strict translation and reverse translation process. Specifically, the content validity was also evaluated through expert review focusing on conceptual and functional equivalence [42, 43]. There were made including real-life and cognitive interviews, some alterations, such as modifying the questionnaire structure, adding additional instructions, and rephrasing questions based on the pretest results. The results obtained through the test–retest method showed the high reliability of the KWCS. In addition, a consistently high level of reliability can be ensured through the sophisticated procedures used in the field investigation and the technical manual provided to the interviewers [44, 45].

Among the KWCS data for care workers, the following data were selected as comparison variables: 1) degree of exposure to work-related risk factors (musculoskeletal and mental), 2) working pattern, 3) work-family balance, 4) work situations, and 5) self-rated health. The tables used for the survey are described in detail in the Additional files (see Supplementary Tables 1-5, Additional files 1, 2, 3, 4, 5 and 6). In this study, missing values ​​were imputed with mean or median or mode values according to data types ​​and group variable were replaced with dummy variable for data analysis.

#### Exposure to work-related musculoskeletal and mental risk factors

The degree of exposure to work-related musculoskeletal and mental risk factors was assessed based on 11 items, including fatigue-inducing or pain-provoking posture, excessive workload, continuous posture, repetitive posture, and dealing with customers. Each item was rated on a seven-point scale, with 1 = all work hours, 2 = nearly all work hours, 3 = ¾ of work hours, 4 = half of work hours, 5 = ¼ of work hours, 6 = almost none, 7 = none at all. In this study, we used the mean score of the 11 items.

#### Working patterns

Working patterns were assessed based on the number of days per month of working overnight at night, on Sunday, Saturday, and for long working hours (10 h <). In this study, we used the average of number of days per month (Additional file 2).

#### Work-family balance

Work-family balance comprised five items about concerns for overtime work and the effect of work intensity on life outside work. Each item was rated on a five-point scale, with 1 = always, 2 = most of the time, 3 = occasionally, 4 = rarely, and 5 = never. A lower score indicated a greater burden of work and poor work-family balance. In this study, we used the mean score of the five items.

#### Work situation

The work situation is a questionnaire to measure organizational justice by asking questions in various situations to explain stress [3]. Work situations were assessed based on 15 items, including supervisors and colleagues, break time at work, stress, decision making, and emotional status. Each item was rated on a five-point scale, with 1 = always, 2 = most of the time, 3 = occasionally, 4 = rarely, and 5 = never. Item number 13 about work stress, and item number 15 about degree of having to hide emotions during work were reverse scored (1: never, 5: always). In this study, we used the mean score for 15 items.

#### Self-health status evaluation

Self-rated health was assessed using a five-point scale, with 1 = very good, 2 = good, 3 = moderate, 4 = poor, and 5 = very poor. In this study, we used the average score for evaluating self-rated health.

#### Work environment satisfaction

Work environment satisfaction is satisfaction with the working conditions of a job. It includes not only the physical environment, but also the organizational culture, relationships with superiors and colleagues, and so on [3]. Each item was rated on a four-point scale, with 1 = very satisfied, 2 = satisfied, 3 = dissatisfied, and 4 = very dissatisfied. In this study, we used the mean score for 15 items.

### Statistical analyses

The results for 1) exposure to work-related musculoskeletal and mental risk factors, 2) work hours, 3) work-family balance, 4) work situations, 5) self-rated health, 6) work environment satisfaction were analyzed to identify differences between caregivers, RNs, and NAs, using one-way ANOVA followed by post-hoc tests and the major predictors of self-rated health and work environment satisfaction were analyzed through stepwise regression model by all variable. SPSS version 18.0 (SPSS Inc., Chicago, IL, USA) was used for all analyses. All statistical significance levels were set at *p* < 0.05.

### Ethical considerations

Ethical approval was not applicable in this study because we utilized secondary data and did not collect or record new information.

## Results

### General characteristics of the participants

As shown in Table 1, of the 494 RNs, 484 were women (98.0%) and 10 were men (2.0%). Of the 201 NAs, 193 were women (96.0%) and eight were men (4.0%). Of the 505 caregivers, 489 were women (96.8%) and 16 were men (3.2%). Ages was rated on a six-point scale, with 1 = 15–19 years old, 2 = 20–29 years old, 3 = 30–39 years old, 4 = 40–49 years old, 5 = 50–59 years old, 6 = 60 years or older. The mean ages were 3.26 ± 0.95 ages among RNs, 3.75 ± 1.00 ages among NAs, and 5.21 ± 0.80 ages among caregivers. The mean work experience was 6.86 ± 6.30 years among RNs, 4.39 ± 4.32 years among NAs, and 4.14 ± 3.38 years among caregivers. Employment status, of the 494 RNs, 484 were full-time (98.0%) and 10 were part-time (2.0%). Of the 201 NAs, 195 were full-time (97.0%) and six were part-time (3.0%). Of the 505 caregivers, 268 were full-time (53.1%) and 237 were part-time (46.9%). Shift work, of the 494 RNs, 176 were yes (64.4%) and 318 were no (35.6%). Of the 201 NAs, 42 were yes (20.9%) and 159 were no (79.1%). Of the 505 caregivers, 118 were yes (21.4%) and 397 were no (78.6%).

**Table 1:** General characteristics of the participants

Variables	Caregiver (A) (*n*=505)	Registered nurse (B) (*n* = 494)	Nurse assistant (C) (*n*=201)
	N(%) or Mean±SD	N(%) or Mean±SD	N(%) or Mean±SD
Gender			
Female	489(96.8)	484(98.0)	193(96,0)
Male	16(3.2)	10(2.0)	8(4.0)
Ages	5.21±0.80	3.26±0.95	3.75±1.00
Work experience (year)	4.14±3.38	6.86±6.30	4.39±4.32
Employment status			
Full-time	268(53.1)	484(98.0)	195(97.0)
Part-time	237(46.9)	10(2.0)	6(3.0)
Shift work			
Yes	118(21.4)	176(35.6)	42(20.9)
No	397(78.6)	318(64.4)	159(79.1)
Education			
No formal education	8(1.6)		
Elementary	50(9.9)		
Middle school	104(20.6)		
High school	299(59.2)	17(3.4)	78(38.8)
College	26(5.1)	193(39.1)	92(45.8)
University	15(3.0)	278(56.3)	30(14.9)
Graduate school	3(0.6)	6(1.2)	1(0.5)

### Comparison of working environment among the caregivers, RNs, and NAs

There were significant differences in work-related musculoskeletal and mental risk exposure, working patterns, work-family balance, work situations, self-rated health, and work environment satisfaction among caregivers, RNs, and NAs (*p* < 0.001).

Post-hoc tests confirmed that the degree of work-related musculoskeletal and mental risk exposure was higher among caregivers and RNs than among NAs, and irregular working patterns, challenges with work-family balance, and work environment satisfaction were higher among caregivers than among NAs. In addition, work situations were poorer among caregivers and NAs than among RNs. Self-rated health was the highest among caregivers, followed by RNs and NAs (Table 2).

**Table 2:** Comparison of working environments among the three groups

Variables	Caregiver (A)	Registered nurse (B)	Nurse assistant (C)	F	p	Post-hoc
Exposure to musculoskeletal and mental work risk factors (score)	4.63 ± 0.75	4.71 ± 0.67	4.97 ± 0.73	27.790	<0.001	A>C, B>C
Working patterns (number/month)	7.92 ± 14.55	8.96 ± 10.52	6.94 ± 9.16	9.279	<0.001	A>C, B>C
Work-family balance (score)	3.53 ± 0.89	3.65 ± 0.73	3.74 ± 0.94	6.712	0.001	A>C
Working situation (score)	2.55 ± 0.48	2.74 ± 0.48	2.61 ± 0.55	10.028	<0.001	A>B, C>B
Self-health status evaluation (score)	2.06 ± 0.62	2.22 ± 0.57	2.44 ± 0.67	45.026	<0.001	A>B>C
Working environment satisfaction (score)	2.14 ± 0.50	2.22 ± 0.48	2.32 ± 0.57	14.911	<0.001	A>C

### Factors affecting self-assessment of health status

The most potent predictor of self-rated health was occupation, followed by work environment satisfaction and work-family balance (Table 3). Furthermore, the most dominant predictor of work environment satisfaction was self-rated health, followed by the degree of exposure to work-related musculoskeletal and mental risk factors, occupation, work-family balance, work situation, and working patterns (Table 4).

**Table 3:** Factors affecting the self-assessment of health status

Variable	B	Partial R^2^	Model R^2^	F	p
Self-assessment of health status (constant)	1.625				
Occupation	0.176	0.068	0.068	89.622	< 0.001
Working environment satisfaction	0.208	0.021	0.099	66.563	< 0.001
Work-family balance	-0.053	0.009	0.108	46.871	< 0.001

**Table 4:** Factors affecting working environment satisfaction

Variable	B	Partial R^2^	Model R^2^	F	p
Working environment satisfaction (constant)	2.135				
Self-assessment of health status	0.139	0.045	0.045	57.250	< 0.001
Exposure to musculoskeletal and mental work risk factors	0.083	0.010	0.055	36.145	< 0.001
Occupation	0.085	0.016	0.071	31.505	< 0.001
Work-family balance	-0.049	0.005	0.076	25.786	< 0.001
Work situation	0.060	0.003	0.079	21.544	< 0.001
Working patterns	0.097	0.002	0.081	18.690	< 0.001

## Discussion

This study aimed to comparatively analyze the work environments of RNs, NAs, and caregivers using the 5^th^ KWCS, to present baseline data for developing occupation-specific measures to improve work environments.

The World Health Organization has emphasized that nursing and care staffs are indispensable healthcare personnel, and that their health is critical to maintaining and promoting the health of mankind [39]. In particular, nursing and care staff work in unique environments with night shifts, exposure to various infectious diseases, excessive workload and tension, and emotional labor [42].

The 3^rd^ KWCS conducted in 2011 analyzed sickness absenteeism among health-related workers and stated that it is important to explore and implement measures to reduce sickness absenteeism for improving health and productivity [39].

The work environment is a comprehensive concept that encompasses the physical workplace, work hours, form of work, and pay. Despite the growing importance of long-term care hospitals and increased demand for more diverse and specialized healthcare services for elderly patients, the work environments for nursing and care staff have not improved [44, 46–48].

In this study, there were significant differences in ages and work environment, namely exposure to work-related musculoskeletal and mental risk factors, working patterns, work-family balance, work situations, self-rated health, and work environment satisfaction among caregivers, RNs, and NAs. Moreover, post-hoc tests confirmed that exposure to work-related musculoskeletal and mental risk factors, and irregular working patterns were higher among caregivers and RNs than among NAs, while challenges with work-family balance, and work environment satisfaction were higher among caregivers than among NAs. Caregivers and NAs were involved in poorer work situations than RNs. Caregivers had the highest self-rated health, followed by RNs and NAs. Thus, despite the fact that caregivers are generally thought of as working in poorer environments than RNs and NAs, they exhibited higher self-rated health and work environment satisfaction. Likewise, the caregivers worked under extremely poor conditions, experiencing anxiety about their position, conflict with healthcare workers, low pay, and labor-intensive work in previous study [8, 9]. This may suggest that since caregivers have relatively less education or expertise compared with RNs and NAs, they may have lower expectations regarding their work environment, or their work intensity may be lower than that of RNs and NAs. Hence, differences in the work environment, intensity, and type of work involved among caregivers, RNs, and NAs should be considered. A previous study also found that organizational management and relationships among members of the organization differed across the types of care-related occupations (nurses and other care workers); consequently, their work-related pressure differed, contributing to the varying response strategies [49]. Moreover, hospitals and nursing homes are predominantly serviced by RNs, NAs, and caregivers through collaborative work, thus requiring continuous measures to improve service quality by implementing occupation-specific measures for enhancing work environment [50].

The factors may have varying impacts on the work environment depending on the occupation. Thus, identifying these factors for each specific occupation is crucial for boosting work performance and ameliorating the work environment [37]. In this study, the strongest predictor of self-rated health was occupation, followed by work environment satisfaction and work-family balance, and the most potent predictor of work environment satisfaction was self-rated health, followed by the degree of exposure to work-related musculoskeletal and mental risk factors, occupation, work-family balance, work situations, and working patterns. As nursing and care staff are the first points of contact who interact with patients most frequently when providing services, they are reported to experience work stress from a number of factors, including excessive workload, insufficient time for nursing and care, irregular working patterns, and poor work environment; exposure to such poor work environments not only adversely impacts their health, but also hinders professional work performance, highlighting a strong association between health status and work environment satisfaction [46].

Thus, it is necessary to foster an environment that promotes health, attributes meaning to work, and facilitates growth among nursing and care staff [51, 52]. A previous study reported that nursing and care staff worked closely with patients and other health professionals, and were predicted to have more difficulties in maintaining a good work-family balance due to work overload caused by short staffing [53], which supports our findings. Also, the work performance of healthcare workers can be influenced by macroscopic factors (health management system) [12], microscopic factors (workplace, facility, and environment) [12], and individual factors (lifestyle and health) [12, 13]. Optimizing performance through macroscopic and microscopic changes, as opposed to individual changes, helps reduce the burden on individual workers, improving their performance [14]. Ameliorating such a work environment is the most efficient method for boosting performance at work [15].

This study analyzed the current problems (e.g., poor work conditions, employment environment, and unpractical staffing standards) in the work environments of nursing and care staff using the 5^th^ KWCS; the findings of this study would be useful as foundational data for developing measures to improve the work environments and conditions for RNs, NAs, and caregivers in preparation for continued population aging. In addition, the results of this study can be commonly applied to East Asian countries (especially Japan and China) that are experiencing the same aging population. Further, the findings may also have implications for relevant systems to ameliorate work environments of RNs, NAs, and caregivers, developing measures to highlight the expertise of staff members and recognize their labor.

The limitations of this study were that only five factors affecting the working environment were selected, and the equivalence of the number of samples and the age of the subjects was not considered. Future studies require more factor-based analyzes and similar sample numbers and age groups, psychological burden and the effects of job title and duties on job satisfaction.

## Conclusion

This study comparatively analyzed the work environments of RNs, NAs, and caregivers using the 5^th^ KWCS to help foster work conditions that promote safe and long-term work among RNs and other care workers in preparation for population aging.

The results showed that there were significant differences not only in the participants’ general characteristics, but also in the degree of exposure to work-related musculoskeletal and mental risk factors, working patterns, work-family balance, work situations, self-rated health, and work environment satisfaction among different care-related occupations. In particular, work environment satisfaction was influenced by an array of factors. Thus, the government and society must devise practical and realistic measures to improve the work environment by considering differences in the environmental features across care-related occupations. Further, longitudinal studies need to be conducted to explore the causative influences among the variables over time, and qualitative studies should analyze the work environments of nursing and other care workers in depth.

## Supplementary Information

**Additional file 1. **Degree of exposure to musculoskeletal and mental occupational risk factors. Table of 11 questionnaires of exposure to musculoskeletal and mental occupational risk factors.

**Additional file 2. **Working patterns. Table of 5 questionnaires of working patterns (evening shift/night shift/Sunday/Saturday/overtime)

**Additional file 3. **Work-life balance. Table of 5 questionnaires about work-life balance

**Additional file 4. **Work situations. Table of 15 questionnaires about Work situations

**Additional file 5. **Self-rated health. Questionnaire about health status, 1: very good, 5: very poor.

**Additional file 6. **Work environment satisfaction. Work environment satisfaction questionnaire, 1: very good, 5: very poor.

## Data Availability

The datasets used and/or analyzed during the current study are available from the corresponding author on reasonable request on reasonable request.

## Abbreviations

RNs: Registered nurses
NAs: Nurse aides
KWCS: Korean Working Conditions Survey
QWL: Quality of work life

## References

[1] Moon S (2015). Recognition of geriatric hospital nurse for medical institution certification, job satisfaction and job stress, study on the turnover intention. J Korean Society Multicultural Health.

[2] Sales A, Sharp N, Li YF, Lowy E, Greiner G, Liu CF (2008). The association between nursing factors and patient mortality in the Veterans Health Administration: the view from the nursing unit level. Med Care.

[3] Oh Y. The 2011 survey on health care level of Koreans. Seoul: Ministry of Health and Welfare. Korea Institute for Health and Social Affairs. https://www.kihasa.re.kr/common/filedown.do.

[4] Byun DS, Yom YH (2009). Factors affecting the burnout of clinical nurses-focused on emotional labor. J Korean Acad Nurs Admin.

[5] Yi M, Oh JH, Hwang HM, Kwon EJ, Lee JH, Park EY (2011). Hospital workers’ experience with hospital evaluation program: a focus group study. J Korean Acad Nurs.

[6] Hwang NM. An analysis of the debates on introduction of public caregivers’ services in acute medical centers. Health Welf Policy Forum. 2010;170:60–71.

[7] Kwak C, Kim SJ, Kang KA, Yim ES (2013). A study on the status and problems of formal caregiving system by hospitals in South Korea. J Korean Data Analysis Soc.

[8] Lee E-H. A study on relationship between job stress and job satisfaction of caretaker’s: base on data gathered from health caretakers of geriatric hospitals*.* Unpublished master's thesis. Seoul: Ewha Woman’s University; 2004.

[9] Kim S (2015). Work and vocational education and training of health care workers in South Korea. Korean Jpn Econ Manag Stud.

[10] Efraty D, Sirgy MJ (1990). The effects of quality of working life (QWL) on employee behavioral responses. Soc Indic Res.

[11] Boonrod W. Quality of working life: perceptions of professional nurses at Phramongkutklao Hospital*.* J Med Assoc Thai. 2009;92(1);Suppl 1:S7–15. 21302412

[12] Dieleman M, Harnmeijer JW (2006). Improving health worker performance: in search of promising practices, (01).

[13] Yun YH, Sim JA, Park EG, Park JD, Noh DY (2016). Employee health behaviors, self-reported health status, and association with absenteeism: comparison with the general population. J Occup Environ Med.

[14] Shekelle PG, Wachter RM, Pronovost PJ, Schoelles K, McDonald KM, Dy SM (2013). Making health care safer II: an updated critical analysis of the evidence for patient safety practices. Evid Rep Technol Assess (Full Rep).

[15] Sagha Zadeh RS, Shepley MM, Owora AH, Dannenbaum MC, Waggener LT, Chung SSE (2018). The importance of specific workplace environment characteristics for maximum health and performance: healthcare workers’ perspective. J Occup Environ Med.

[16] Massachusetts Hospital Association (2010). Hospital costs in context: a transparent view of the cost of care.

[17] Kirk SJ, Dell’Isola AJ. Life cycle costing for design professionals. 1995.

[18] Page A, Committee on the Work Environment for Nurses, Patient Safety Board on Health Care Services. Institute of Medicine: Keeping Patients Safe-Transforming the Work Environment of Nurses; 2004.

[19] Goodman PS. Missing organizational linkages: Tools for cross-level research. California: Sage; 2000.

[20] Reiling J, Hughes RG, Murphy MR (2008). The impact of facility design on patient safety*.* Patient safety and quality: an evidence-based handbook for nurses.

[21] Meenakshi MS, Ravichandran K, CV MV (2013). Quality of work life–the need of the hour. Int J Bus Manag Invent..

[22] Cole DC, Robson LS, Lemieux-Charles L, McGuire W, Sicotte C, Champagne F (2005). Quality of working life indicators in Canadian health care organizations: a tool for healthy, health care workplaces?. Occup Med (Lond).

[23] Hosseinabadi R, Karampourian A, Beiranvand S, Pournia Y (2013). The effect of quality circles on job satisfaction and quality of work-life of staff in emergency medical services. Int Emerg Nurs.

[24] Hsu MY, Kernohan G (2006). Dimensions of hospital nurses’ quality of working life. J Adv Nurs.

[25] Almalki MJ, FitzGerald G, Clark M (2012). The relationship between quality of work life and turnover intention of primary health care nurses in Saudi Arabia. BMC Health Serv Res.

[26] An JY, Yom YH, Ruggiero JS (2011). Organizational culture, quality of work life, and organizational effectiveness in Korean university hospitals. J Transcult Nurs.

[27] Hornung S, Glaser J, Rousseau DM, Angerer P, Weigl M (2011). Employee-oriented leadership and quality of working life: mediating roles of idiosyncratic deals. Psychol Rep.

[28] Beasley JW, Karsh BT, Sainfort F, Hagenauer ME, Marchand L (2004). Quality of work life of family physicians in Wisconsin’s health care organizations: a WReN study. Wis Med J.

[29] Beasley JW, Karsh BT, Hagenauer ME, Marchand L, Sainfort F (2005). Quality of work life of independent *vs* employed family physicians in Wisconsin: a WReN study. Ann Fam Med.

[30] Minvielle E, Sicotte C, Champagne F, Contandriopoulos AP, Jeantet M, Préaubert N (2008). Hospital performance: competing or shared values?. Health Policy.

[31] Nayeri ND, Salehi T, Noghabi AA (2011). Quality of work life and productivity among Iranian nurses. Contemp Nurse.

[32] Phan GT, Vo TQ (2016). A literature review on quality of working life: a case of healthcare workers. J App Pharm Sci.

[33] Klopper HC, Coetzee SK, Pretorius R, Bester P (2012). Practice environment, job satisfaction and burnout of critical care nurses in South Africa. J Nurs Manag.

[34] Broadbent DE (1971). Decision and stress.

[35] Broadbent DE (1979). Human performance and noise. Handbook of noise control.

[36] Trinkoff AM, Le R, Geiger-Brown J, Lipscomb J, Lang G (2006). Longitudinal relationship of work hours, mandatory overtime, and on-call to musculoskeletal problems in nurses. Am J Ind Med.

[37] Hajizadeh A, Zamanzadeh V, Kakemam E, Bahreini R, Khodayari-Zarnaq R (2021). Factors influencing nurses participation in the health policy-making process: A systematic review. BMC Nurs.

[38] Park S, Park C, Sung JH (2022). How does the involuntary choice of self-employment affect subjective well-being in small-sized business workers? A cross-sectional study from the fifth Korean working conditions survey. Int J Environ Res Public Health.

[39] Kim J, Yu J (2020). Impact of nurses’ occupational environment on preseentism. Glob Health Nurs.

[40] Kim YS, Rhee KY, Oh MJ, Park J (2013). The validity and reliability of the second Korean working conditions survey. Saf Health Work.

[41] Choi M, Suh C, Choi SP, Lee CK, Son BC (2020). Validation of the work engagement scale-3, used in the 5th Korean working conditions survey. Annals of occupational and environmental medicine.

[42] Kim HS, Kim KH. The study of work environment of nurses in long-term care hospitals*.* J Korea Academia-Industrial Cooperation Soc. 2019;20:250–8.

[43] Korea Occupational Safety & Health Agency. The 5th Working Environment Survey. Survey Development and Supplementary Report. 2017. Available online: https://www.kosha.or.kr/kosha/data/registration.do?mode=view&articleNo=327746&article.offset=0&articleLimit=10#/list. Accessed 7 Nov 2022.

[44] Min A, Kang M, Hong HC. Sickness presenteeism in shift and non-shift nurses: using the fifth Korean working conditions survey. Int J Environ Res Public Health. 2021;18(6):3236. 10.3390/ijerph18063236 PMC8004057 33800982

[45] Kim JK. A review study on nursing students' stress for improvement of nursing education. J Korean Academic Soc Nurs Educ. 2014;20:47–59.

[46] Jang H, Choi E. The impact of nurses’ working environment on health problems. Korean J Occup Health Nurs. 2020;29:1–7.

[47] Ahn SH, Jung SH, You JH, Lee M (2018). Nursing tasks and practice environment for nursing work perceived by nurses working on comprehensive wards *versus* general wards. J Korean Acad Nurs Adm.

[48] Choi ES, Jeon GS (2017). The impacts of psychosocial work environments on depressive symptoms among Korean registered nurses. Korean J Occup Health Nurs.

[49] Button LA (2008). Effect of social support and coping strategies on the relationship between health care-related occupational stress and health. J Res Nurs.

[50] Yeun YR (2015). Effects of comprehensive nursing service on the nursing performance, job satisfaction and customer orientation among nurses. J Korea Acad-Ind Coop Soc.

[51] June KJ, Choi ES, Park MJ (2013). Effect of psychosocial work environment and self-efficacy on mental health of office workers. Korean J Occup Health Nurs.

[52] Aust B, Rugulies R, Skakon J, Scherzer T, Jensen C (2007). Psychosocial work environment of hospital workers: validation of a comprehensive assessment scale. Int J Nurs Stud.

[53] Grzywacz JG, Frone MR, Brewer CS, Kovner CT (2006). Quantifying work–family conflict among registered nurses. Res Nurs Health.

