# Supplementary material for: A comparative analysis of the work environments for registered nurses, nurse aides, and caregivers using the 5th Korean Working Conditions Survey
Source: BMC Nurs. 2022 Dec 13;21:356. doi: 10.1186/s12912-022-01120-9 (PMC9746153; doi:10.1186/s12912-022-01120-9)
Supplement: Supplementary file 1 — Additional file 1. Degree of exposure to musculoskeletal and mental occupational risk factors. Table of 11 questionnaires of exposure to musculoskeletal and mental occupational risk factors. [file 12912_2022_1120_MOESM1_ESM.doc]

Supplementary Table 1. Degree of exposure to musculoskeletal and mental occupational risk factors

| Name of variable | Explanation of variable | Content |
| --- | --- | --- |
| Q26.  Degree of exposure to musculoskeletal and mental occupational risk factors | A. Degree to which the job involves fatigue-inducing or pain-provoking posture | 1. Entire work hours 2. Nearly entire work hours  3. ¾ of work hours 4. Half of work hours  5. ¼ of work hours 6. Almost none  7. None at all 8. I do not know/no response  9. Decline to answer |
|  | B. Degree to which the job involves lifting or moving a person | 1. Entire work hours 2. Nearly entire work hours  3. ¾ of work hours 4. Half of work hours  5. ¼ of work hours 6. Almost none  7. None at all 8. I do not know/no response  9. Decline to answer |
|  | C. Degree to which the job involves pulling, pushing, or moving heavy objects | 1. Entire work hours 2. Nearly entire work hours  3. ¾ of work hours 4. Half of work hours  5. ¼ of work hours 6. Almost none  7. None at all 8. I do not know/no response  9. Decline to answer |
|  | D. Degree to which the job involves continuous standing | 1. Entire work hours 2. Nearly entire work hours  3. ¾ of work hours 4. Half of work hours  5. ¼ of work hours 6. Almost none  7. None at all 8. I do not know/no response  9. Decline to answer |
|  | E. Degree to which the job involves sedentary posture | 1. Entire work hours 2. Nearly entire work hours  3. ¾ of work hours 4. Half of work hours  5. ¼ of work hours 6. Almost none  7. None at all 8. I do not know/no response  9. Decline to answer |
|  | F. Degree to which the job involves repetitive hand or arm motions | 1. Entire work hours 2. Nearly entire work hours  3. ¾ of work hours 4. Half of work hours  5. ¼ of work hours 6. Almost none  7. None at all 8. I do not know/no response  9. Decline to answer |
|  | G. Degree to which the job involves directly dealing with people other than colleagues, such as clients, passengers, students, and patients | 1. Entire work hours 2. Nearly entire work hours  3. ¾ of work hours 4. Half of work hours  5. ¼ of work hours 6. Almost none  7. None at all 8. I do not know/no response  9. Decline to answer |
|  | H. Degree to which the job involves dealing with angry clients, patients, and students | 1. Entire work hours 2. Nearly entire work hours  3. ¾ of work hours 4. Half of work hours  5. ¼ of work hours 6. Almost none  7. None at all 8. I do not know/no response  9. Decline to answer |
|  | I. Degree to which the job involves the use of a computer, laptop, or smartphone | 1. Entire work hours 2. Nearly entire work hours  3. ¾ of work hours 4. Half of work hours  5. ¼ of work hours 6. Almost none  7. None at all 8. I do not know/no response  9. Decline to answer |
|  | J. Degree to which the job involves the use of the internet and emails | 1. Entire work hours 2. Nearly entire work hours  3. ¾ of work hours 4. Half of work hours  5. ¼ of work hours 6. Almost none  7. None at all 8. I do not know/no response  9. Decline to answer |
|  | K. Degree to which the job involves being placed in an emotionally unsettling situation | 1. Entire work hours 2. Nearly entire work hours  3. ¾ of work hours 4. Half of work hours  5. ¼ of work hours 6. Almost none  7. None at all 8. I do not know/no response  9. Decline to answer |
