# Supplementary material for: A comparative analysis of the work environments for registered nurses, nurse aides, and caregivers using the 5th Korean Working Conditions Survey
Source: BMC Nurs. 2022 Dec 13;21:356. doi: 10.1186/s12912-022-01120-9 (PMC9746153; doi:10.1186/s12912-022-01120-9)
Supplement: Supplementary file 2 — Additional file 2. Working patterns. Table of 5 questionnaires of working patterns (evening shift/night shift/Sunday/Saturday/overtime) [file 12912_2022_1120_MOESM2_ESM.doc]

Supplementary Table 2. Working patterns

(Graveyard shift/night shift/Sunday/Saturday/overtime)

| Working patterns | A. Number of night shifts per month | □□ days a month  0. None  88. I do not know/no response 99. Decline to answer |
| --- | --- | --- |
|  | B. Number of evening shifts per month | □□ days a month  0. None  88. I do not know/no response 99. Decline to answer |
|  | C. Number of Sunday shifts per month | □□ days a month  0. None  88. I do not know/no response 99. Decline to answer |
|  | D. Number of Saturday shifts per month | □□ days a month  0. None  88. I do not know/no response 99. Decline to answer |
|  | E. Number of days working overtime per month | □□ days a month  0. None  88. I do not know/no response 99. Decline to answer |
