# Supplementary material for: A comparative analysis of the work environments for registered nurses, nurse aides, and caregivers using the 5th Korean Working Conditions Survey
Source: BMC Nurs. 2022 Dec 13;21:356. doi: 10.1186/s12912-022-01120-9 (PMC9746153; doi:10.1186/s12912-022-01120-9)
Supplement: Supplementary file 3 — Additional file 3. Work-life balance. Table of 5 questionnaires about work-life balance [file 12912_2022_1120_MOESM3_ESM.doc]

Supplementary Table 3. Work-family balance

| Work-family balance | A. I worry about work even when I am not working | 1. Always 2. Most of the time  3. Occasionally 4. Rarely  5. Never  7. Not applicable  8. I do not know/no response 9. Decline to answer |
| --- | --- | --- |
|  | B. I cannot do any housework after coming home from work because I am too tired | 1. Always 2. Most of the time  3. Occasionally 4. Rarely  5. Never  7. Not applicable  8. I do not know/no response 9. Decline to answer |
|  | C. I do not have enough time to spend with my family due to my work | 1. Always 2. Most of the time  3. Occasionally 4. Rarely  5. Never  7. Not applicable  8. I do not know/no response 9. Decline to answer |
|  | D. I do not have enough time for work because of things that happen at home | 1. Always 2. Most of the time  3. Occasionally 4. Rarely  5. Never  7. Not applicable  8. I do not know/no response 9. Decline to answer |
|  | E. I feel like I cannot work as much as I need to because of my responsibilities for my family. | 1. Always 2. Most of the time  3. Occasionally 4. Rarely  5. Never  7. Not applicable  8. I do not know/no response 9. Decline to answer |
