# Supplementary material for: A comparative analysis of the work environments for registered nurses, nurse aides, and caregivers using the 5th Korean Working Conditions Survey
Source: BMC Nurs. 2022 Dec 13;21:356. doi: 10.1186/s12912-022-01120-9 (PMC9746153; doi:10.1186/s12912-022-01120-9)
Supplement: Supplementary file 4 — Additional file 4. Work situations. Table of 15 questionnaires about Work situations [file 12912_2022_1120_MOESM4_ESM.doc]

Supplementary Table 4. Work situations

| Work situations | A. My colleagues help and support me | 1. Always 2. Most of the time  3. Occasionally 4. Rarely  5. Never  7. Not applicable  8. I do not know/no response 9. Decline to answer |
| --- | --- | --- |
|  | B. My boss helps and supports me | 1. Always 2. Most of the time  3. Occasionally 4. Rarely  5. Never  7. Not applicable  8. I do not know/no response 9. Decline to answer |
|  | C. They ask me for my thoughts before my work goals are decided | 1. Always 2. Most of the time  3. Occasionally 4. Rarely  5. Never  7. Not applicable  8. I do not know/no response 9. Decline to answer |
|  | D. I participate in improving the work groups or processes at my department or organization | 1. Always 2. Most of the time  3. Occasionally 4. Rarely  5. Never  7. Not applicable  8. I do not know/no response 9. Decline to answer |
|  | E. My opinions are reflected when choosing people to work with | 1. Always 2. Most of the time  3. Occasionally 4. Rarely  5. Never  7. Not applicable  8. I do not know/no response 9. Decline to answer |
|  | F. I am able to take a break when I want to | 1. Always 2. Most of the time  3. Occasionally 4. Rarely  5. Never  7. Not applicable  8. I do not know/no response 9. Decline to answer |
|  | G. I have enough time to complete my work | 1. Always 2. Most of the time  3. Occasionally 4. Rarely  5. Never  7. Not applicable  8. I do not know/no response 9. Decline to answer |
|  | H. I feel like I have done a good job when I work | 1. Always 2. Most of the time  3. Occasionally 4. Rarely  5. Never  7. Not applicable  8. I do not know/no response 9. Decline to answer |
|  | I. I can apply my thoughts to my work | 1. Always 2. Most of the time  3. Occasionally 4. Rarely  5. Never  7. Not applicable  8. I do not know/no response 9. Decline to answer |
|  | J. I feel like I am doing something worthy | 1. Always 2. Most of the time  3. Occasionally 4. Rarely  5. Never  7. Not applicable  8. I do not know/no response 9. Decline to answer |
|  | K. I know what is expected of me at work | 1. Always 2. Most of the time  3. Occasionally 4. Rarely  5. Never  7. Not applicable  8. I do not know/no response 9. Decline to answer |
|  | L. I am treated fairly at work | 1. Always 2. Most of the time  3. Occasionally 4. Rarely  5. Never  7. Not applicable  8. I do not know/no response 9. Decline to answer |
|  | M. I am stressed from work | 1. Always 2. Most of the time  3. Occasionally 4. Rarely  5. Never  7. Not applicable  8. I do not know/no response 9. Decline to answer |
|  | N. I have a say in important decision-making at work | 1. Always 2. Most of the time  3. Occasionally 4. Rarely  5. Never  7. Not applicable  8. I do not know/no response 9. Decline to answer |
|  | O. I need to hide my emotions while working | 1. Always 2. Most of the time  3. Occasionally 4. Rarely  5. Never  7. Not applicable  8. I do not know/no response 9. Decline to answer |
