# Supplementary material for: A comparative analysis of the work environments for registered nurses, nurse aides, and caregivers using the 5th Korean Working Conditions Survey
Source: BMC Nurs. 2022 Dec 13;21:356. doi: 10.1186/s12912-022-01120-9 (PMC9746153; doi:10.1186/s12912-022-01120-9)
Supplement: Supplementary file 5 — Additional file 5. Self-rated health. Questionnaire about health status, 1: very good, 5: very poor. [file 12912_2022_1120_MOESM5_ESM.doc]

Supplementary Table 5. Self-rated health

| Self-rated health | Health status | 1. Very good 2. Good  3. Moderate 4. Poor  5. Very poor 8. I do not know/no response  9. Decline to answer |
| --- | --- | --- |
