# Supplementary material for: A comparative analysis of the work environments for registered nurses, nurse aides, and caregivers using the 5th Korean Working Conditions Survey
Source: BMC Nurs. 2022 Dec 13;21:356. doi: 10.1186/s12912-022-01120-9 (PMC9746153; doi:10.1186/s12912-022-01120-9)
Supplement: Supplementary file 6 — Additional file 6. Work environment satisfaction. Work environment satisfaction questionnaire, 1: very good, 5: very poor. [file 12912_2022_1120_MOESM6_ESM.doc]

Supplementary Table 6. Work environment satisfaction

| Q 69 | Work environment satisfaction | 1. Very satisfied  2. Satisfied  3. Not really satisfied  4. Very dissatisfied  8. I do not know/no response 9. Decline to answer |
| --- | --- | --- |
